# Genome-Wide Identification of the Wheat *GAD* Gene Family Reveals TaGAD1-Mediated Salt Tolerance via GABA-Dependent ROS Homeostasis

**DOI:** 10.3390/plants15152281

**Published:** 2026-07-25

**Authors:** Xingbei Liu, Ming Wang, Jiajia Zhou, Yan Li, Guoli Li, Shengran He, Jixi Li, Xiang Huang, Jinyan Cheng, Gui Wang, Haifeng Guo, Jinpeng Li, Qijin Lou

**Affiliations:** 1 College of Resources and Environment, Moutai Institute, Renhuai 564502, China; liuxingbei1991@163.com (X.L.);; 2 China State Key Laboratory of Crop Genetics & Germplasm Enhancement and Utilization, College of Agriculture, Nanjing Agricultural University, Nanjing 210095, China; 3 College of Agronomy and Biotechnology, China Agricultural University, Beijing 100193, China

**Keywords:** wheat, glutamate decarboxylase (GAD), genome-wide identification, salt stress

## Abstract

Wheat (*Triticum aestivum* L.) is a major food crop that is severely affected by salt stress, resulting in significant yield losses. Glutamic acid decarboxylase (GAD) catalyzes the irreversible conversion of glutamic acid to γ-aminobutyric acid (GABA) and plays key roles in plant growth, development, and stress responses. However, the GAD gene family in hexaploid wheat and its role in salt tolerance remain poorly understood. In this study, the wheat GAD gene family was systematically identified. Genome-wide analysis revealed seven *TaGAD* genes with 19 gene copies. A Ka/Ks ratio < 1 indicates strong evolutionary conservation of this family. All TaGAD proteins contain a conserved Glu-decarb-GAD domain with similar motif composition and structural organization. Promoter analysis showed enrichment of stress-responsive cis-elements. Expression profiling demonstrated tissue-specific patterns, with several *TaGAD* genes significantly induced under salt stress. CRISPR/Cas9-mediated knockout of *TaGAD1* led to markedly reduced salt tolerance, accompanied by decreased GABA content and GAD activity, reduced activities of antioxidant enzymes (SOD, POD, and CAT), and excessive accumulation of reactive oxygen species (ROS). These results demonstrate that *TaGAD1* positively regulates salt tolerance in wheat through GABA-mediated ROS homeostasis. This study provides a systematic characterization of the wheat *TaGAD* gene family in the context of salt stress, laying a theoretical foundation for understanding GABA-mediated tolerance mechanisms and identifying *TaGAD1* as a potential molecular breeding target for improving salt tolerance in wheat.

## 1. Introduction

Soil salinization is one of the most severe abiotic stresses limiting global crop productivity and poses a major threat to food security, particularly for staple cereals such as wheat (*Triticum aestivum* L.) [1,2]. More than 800 million hectares of land worldwide are affected by salinity, with irrigated agricultural systems being especially prone to secondary salinization [1,3]. Wheat accounts for approximately 20% of the world’s caloric intake [4,5]; however, salinity can reduce the wheat yield by 20–50%, depending on stress intensity and duration [1]. Under future climate change scenarios, these losses are likely to become even more severe, emphasizing the urgent need to develop salt-tolerant wheat cultivars [6].

The harmful effects of salt stress on plants are complex and multifactorial, encompassing osmotic stress that impairs water uptake, ion toxicity caused mainly by the excessive accumulation of Na^+^ and Cl^−^, and oxidative stress resulting from the overproduction of reactive oxygen species (ROS) [1,2,7]. Plant responses to salinity generally occur in two phases: an initial osmotic phase that rapidly suppresses the growth of young leaves, and a subsequent ionic phase that accelerates the senescence of mature leaves [1]. Plant strategies for adapting to salinity can be broadly classified into three categories: osmotic stress tolerance, Na^+^ or Cl^−^ exclusion, and tissue tolerance to accumulated Na^+^ or Cl^−^ [1]. These strategies are underpinned by distinct molecular mechanisms, among which ion homeostasis and ROS detoxification are of particular importance.

To maintain Na^+^ homeostasis under saline conditions, plants have evolved a range of ion transport systems. The salt overly sensitive (SOS) pathway constitutes the central signaling cascade governing Na^+^ exclusion, in which the SOS3/SOS2 kinase complex phosphorylates and activates the plasma membrane Na^+^/H^+^ antiporter SOS1, thereby promoting Na^+^ efflux from root epidermal cells [8]. In addition, SOS1 is also involved in long-distance Na^+^ transport by facilitating Na^+^ retrieval from the xylem under severe salt stress [9]. The HKT gene family further contributes to Na^+^ exclusion from aerial tissues by mediating Na^+^ unloading from the xylem into surrounding parenchyma cells, as exemplified by AtHKT1 in *Arabidopsis* [10] and TmHKT1;5-A/Nax2 in wheat, which has been reported to increase the grain yield by 25% under field saline conditions [11]. Vacuolar Na^+^ sequestration mediated by NHX antiporters, such as AtNHX1, constitutes another crucial mechanism for alleviating cytosolic Na^+^ toxicity [12]. In wheat, the K^+^/H^+^ exchanger TaNHX2 has been shown to interact with TaGAD1, thereby enhancing drought tolerance through stomatal regulation and GABA accumulation [13], highlighting the emerging functional interplay between ion transporters and GABA metabolism.

In parallel with ion homeostasis, maintaining ROS homeostasis is equally essential for salt tolerance. Under salt stress, excessive ROS—including H_2_O_2_, O_2_^−^, and hydroxyl radicals—cause damage to lipids, proteins, and nucleic acids, leading to membrane destabilization and cell death [7]. Plants deploy both enzymatic (SOD, CAT, APX, GR, GPX, GST) and non-enzymatic (ascorbic acid, glutathione, α-tocopherols) antioxidant systems to scavenge ROS [7]. However, ROS also serve as important signaling molecules that regulate the expression of stress-responsive genes and modulate growth, cell cycle, and programmed cell death [14]. The dual role of ROS—both destructive and regulatory—necessitates tight coordination between ROS production and scavenging, a balance [15]. Several transcription factors have been shown to integrate ROS detoxification with salt tolerance in crops; for example, TaWRKY17 and TaWRKY44 synergistically promote the expression of the dehydrin gene *TaDHN7* to enhance ROS scavenging in wheat [16], and HvCBP60-8 in barley activates *HvHKT1;1*, *HvNHX1*, and *HvHAK1* to maintain K^+^/Na^+^ homeostasis under salt stress [17].

Among the metabolites that bridge ion homeostasis, ROS regulation, and stress signaling, γ-aminobutyric acid (GABA) has emerged as a key player. GABA, a four-carbon non-protein amino acid, is a major constituent of the free amino acid pool in most prokaryotic and eukaryotic organisms [18]. GABA is involved in pH regulation, C/N balance, plant development and defense, as well as functioning as a compatible osmolyte [18]. Beyond its metabolic roles, GABA serves as a signaling molecule that directly regulates the activity of plant-specific anion transporters, including ALMT proteins, thereby modulating root growth and tolerance to acidic pH and aluminum ions [19]. Accumulating evidence indicates that GABA functions not only as an osmo-protectant, but also as a signaling molecule that modulates ion transport, ROS metabolism, and the expression of stress-responsive genes [20]. In tomato (*Solanum lycopersicum*), exogenous GABA treatment significantly reduced Na^+^ accumulation in leaves and roots under salt stress, decreased Na^+^ influx and root-to-shoot transport, and upregulated the expression of *SlGAD1–SlGAD3*, thereby enhancing GAD activity and endogenous GABA accumulation, ultimately alleviating salt-induced damage [21]. In Arabidopsis, GABA enhances salt tolerance by activating plasma membrane H^+^-ATPase, promoting cytosolic K^+^ retention, reducing Na^+^ influx, and upregulating *SOS1* and *NHX1* expression [22], whereas disruption of the GABA shunt in the *pop2-1* mutant impairs central carbon metabolism and compromises salt tolerance [23]. In wheat, a recent study demonstrated that exogenous GABA application alleviates salt-induced growth inhibition by protecting the photosynthetic system (as indicated by increased chlorophyll fluorescence parameters including Fv/Fm, Y(II), qP, and ETRmax), maintaining Na^+^/K^+^ homeostasis, and reducing oxidative damage, while the silencing of *TaGAD6* was shown to compromise salt tolerance [24]. These findings collectively suggest that GABA operates at the intersection of ion transport and ROS homeostasis to facilitate salt adaptation.

GABA is synthesized primarily via the GABA shunt, in which glutamate decarboxylase (GAD, EC 4.1.1.15) catalyzes the irreversible decarboxylation of L-glutamate to produce GABA and CO_2_ [18]. GAD is thus the rate-limiting enzyme of the GABA shunt, and its activity is regulated by Ca^2+^/calmodulin (CaM) binding to a conserved C-terminal autoinhibitory domain [25]. The Ca^2+^/CaM-dependent activation of GAD provides a direct molecular link between calcium signaling and GABA accumulation, enabling rapid GABA synthesis in response to stress-induced calcium transients [26]. In wheat, the K^+^/H^+^ exchanger TaNHX2 has been shown to target the C-terminal autoinhibitory domain of TaGAD1, enhancing its activity and promoting GABA accumulation under drought stress [13], suggesting that GAD activity can be modulated not only by Ca^2+^/CaM, but also through protein–protein interactions with ion transporters.

The gene families involved in GABA metabolism have been systematically characterized in multiple plant species. In cotton (*Gossypium hirsutum*), members of the GAD, GABA-T, and SSADH families exhibit differential expression patterns under salt and drought stresses, and GABA application enhances leaf antioxidant enzyme activities while reducing ROS accumulation [27]. In apple (*Malus domestica*), GABA pathway gene families show pronounced tissue-specific expression, providing a foundation for exploring their functional divergence in development and stress responses [28]. A genome-wide analysis of the GABA pathway gene families in walnut (*Juglans regia*) identified the 5 *GAD*, 9 *GABA-T*, and 25 *SSADH* genes, and expression profiling revealed that specific members (particularly *JrSSADH23*) are significantly upregulated under salt and drought stress [29]. However, despite the growing understanding of GABA metabolism in model and crop species, the *GAD* gene family in wheat—the world’s most widely cultivated cereal—has not been systematically identified, and the functional role of specific TaGAD members in salt tolerance remains unexplored. Notably, while TaGAD1 has been implicated in drought tolerance through its interaction with TaNHX2 [13], its contribution to salt tolerance and the underlying mechanism have not been directly investigated.

In this study, we systematically identified the *GAD* gene family in hexaploid wheat through genome-wide analysis, characterized their evolutionary relationships, gene structures, conserved motifs, chromosomal distribution, and cis-element composition, and analyzed their tissue-specific and stress-responsive expression patterns. Furthermore, we functionally characterized *TaGAD1* using CRISPR/Cas9-mediated knockout to determine its role in salt tolerance and explored whether TaGAD1 regulates salt tolerance through GABA-mediated ROS detoxification. Our findings provide the first direct genetic evidence linking a specific GAD gene to salt tolerance in wheat and establish TaGAD1 as a promising candidate for molecular breeding aimed at improving salt tolerance in this major staple crop.

## 2. Materials and Methods

### 2.1. Plant Materials and Culture Conditions

The wheat (*Triticum aestivum* L.) cultivar ‘Fielder’ was used as the wild-type (WT) control and as the genetic background for all CRISPR/Cas9-derived mutant lines. Seeds were surface-sterilized with 75% (*v*/*v*) ethanol for 5 min, followed by 2% (*v*/*v*) sodium hypochlorite for 15 min, rinsed thoroughly with sterile distilled water, and stratified at 4 °C for 3 days in the dark.

### 2.2. Plant Growth and Salt Treatment

Germinated seeds were transferred to a hydroponic system and grown in half-strength Hoagland nutrient solution under a 12 h light/12 h dark photoperiod with cool-white fluorescent light (3000 lux) at 26/20 °C (day/night) and 60% relative humidity. Seven-day-old hydroponically grown seedlings were treated with 0 mM (control) or 250 mM NaCl solution for 7 days. The nutrient solution was renewed every 2–3 days to maintain stable NaCl concentrations and nutrient supply.

At the end of the treatment, shoot fresh weight and dry weight were recorded. For dry weight determination, shoot tissues were dried at 80 °C for 48 h to constant weight. At least three biological replicates were performed for each experiment.

### 2.3. Identification of Wheat GAD Gene Family Members

To identify members of the *GAD* gene family in wheat (*T. aestivum*), the whole-genome sequence and annotation files of wheat were retrieved from the Ensembl Plants database (http://plants.ensembl.org). The hidden Markov model (HMM) profile corresponding to the pyridoxal phosphate-dependent decarboxylase domain (PF00282) was obtained from the Pfam database (http://pfam.xfam.org).

The amino acid sequences of five *Arabidopsis thaliana* GAD proteins (AtGAD1–AtGAD5) were used as queries for BLASTp searches against the wheat proteome with an E-value threshold of 1 × 10^−5^. Redundant sequences were removed manually. The whole genome sequence of wheat was retrieved from the Ensembl Plants database (http://plants.ensembl.org). The hidden Markov model (HMM) profile corresponding to the pyridoxal phosphate-dependent decarboxylase domain (PF00282) was obtained from the Pfam database (http://pfam.xfam.org). Candidate protein sequences were initially screened using HMMER (version 3.0) with the pyridoxal phosphate-dependent decarboxylase domain HMM as a query, followed by domain validation via the SMART (http://smart.embl.de) (accessed on 8 August 2025) and NCBI Conserved Domain Database (CDD) tools (https://www.ncbi.nlm.nih.gov/cdd/) (accessed on 8 August 2025). The amino acid sequence of wheat *TaGADs* was analyzed for its length, isoelectric point, molecular weight, and other physicochemical properties using the ExPASy website (https://web.expasy.org/protparam/) (accessed on 19 August 2025).

### 2.4. Gene Structure and Conserved Motif Analysis

The exon–intron structures of *TaGAD* genes were visualized using the Gene Structure Display Server (GSDS 2.0; http://gsds.gao-lab.org/) by comparing the coding sequences (CDS) with their corresponding genomic sequences. Conserved protein motifs in TaGAD sequences were identified using the MEME Suite (version 5.0.2; https://meme-suite.org/meme/ (accessed on 19 August 2025)). Conserved domains were annotated using the NCBI CDD and Pfam databases.

### 2.5. Evolution of the GAD Gene Family

The whole genome sequence of maize (*Zea mays*), wheat (*Triticum aestivum*), rice (*Oryza sativa*), Brachypodium (*Brachypodium distachyon*), and Arabidopsis (*Arabidopsis thaliana*) was retrieved from the Ensembl Plants database (http://plants.ensembl.org). Multiple sequence alignment of GAD amino acid sequences was performed with ClustalX (version 2.0) using default parameters, followed by evolutionary tree construction in MEGA7.0 using the neighbor-joining method [30] (Poisson correction, pairwise deletion, 1000 bootstrap replicates). The phylogenetic tree was meticulously annotated and aesthetically refined using Evolview (https://www.evolgenius.info/evolview/) (accessed on 2 Steptember 2025) [31], an online platform.

### 2.6. Protein Structure Prediction

SOPMA (http://npsa-pbil.ibcp.fr/) (accessed on 20 Steptember 2025) and SWISS-MODLE (https://swissmodel.expasy.org/) (accessed on 28 Steptember 2025) [32] were used to predict the secondary and tertiary structures of proteins, respectively.

The secondary structures of TaGAD proteins were predicted using SOPMA (Self-Optimized Prediction Method with Alignment; https://npsa-prabi.ibcp.fr/cgi-bin/npsa_automat.pl?page=/NPSA/npsa_sopma.html) (accessed on 20 Steptember 2025). Tertiary structure models were generated using the SWISS-MODEL server (https://swissmodel.expasy.org/) (accessed on 28 Steptember 2025) with default parameters, selecting the template with the highest GMQE (Global Model Quality Estimation) score. Structural classification of TaGAD proteins was based on the similarity of their predicted tertiary structures.

### 2.7. Chromosome Localization and Synteny Analysis of the TaGAD Gene Family

The chromosomal location information of *TaGAD* genes was retrieved from the wheat genome annotation file and visualized using the online tool MG2C v2.1 (http://mg2c.iask.in/mg2c_v2.1/) (accessed on 25 January 2026) [33].

Collinearity analysis within the wheat genome and between wheat and other species (rice, maize, *Brachypodium distachyon*, and *Arabidopsis thaliana*) was performed and visualized using TBtools-II (v2.154) [34]. Genome-wide data and annotation files for other species were obtained from the Ensembl Plants database (https://plants.ensembl.org/index.html).

The nonsynonymous/synonymous substitution ratio (Ka/Ks) for homologous gene pairs was calculated using the Simple Ka/Ks Calculator module in TBtools [34].Gene pairs with Ka/Ks < 1 were considered under purifying selection, Ka/Ks = 1 under neutral evolution, and Ka/Ks > 1 under positive selection [35].

### 2.8. Analysis of Cis-Acting Elements of TaGAD Gene Family

The DNA sequences of the 2000-bp upstream region from the translation start codon (ATG) of each *TaGAD* gene were extracted from the wheat whole-genome database [36].

Cis-acting elements in these promoter regions were analyzed using the PlantCARE database (https://bioinformatics.psb.ugent.be/webtools/plantcare/html/) (accessed on 30 January 2026) [37], and the results were subsequently visualized using TBtools.

### 2.9. RNA Extraction, RT-qPCR, and Transcriptome Data Analysis

Seven-day-old wheat seedlings were subjected to heat, drought, and salt stress treatments, respectively. For heat stress, seedlings were placed in a growth chamber at 42 °C for 1, 2, and 3 h, and then sampled immediately. For drought stress, seedlings were exposed to air for 1, 2, and 3 h to induce natural dehydration, following a previously reported method [38]. For salt stress, seedlings were treated with 150 mM NaCl solution for 0, 1, 3, 6, 9, and 12 h. Following each treatment, total RNA was extracted from samples using Trizol (Vazyme Biotech Co., Ltd., Nanjing, China, R411), and first-strand cDNAs were synthesized from 1 μg starting total RNA using a reverse transcription kit (Vazyme Biotech, R223-01) according to the manufacturer’s instructions. Real-time quantitative PCR (qPCR) was performed with Taq Pro Universal SYBR qPCR Master Mix (Vazyme) on a C1000 Touch Real-Time PCR system (Bio-Rad, Santa Clara, CA, USA) to detect specific genes. The primers used for RT-qPCR are listed in Table S1. Differences in relative transcript levels were calculated using the 2^–ΔCT^ method. All RT-qPCR experiments were performed with three biological replicates.

The tissue-specific expression patterns of *TaGAD* genes were analyzed using the publicly available strand-specific time-series transcriptome atlas of wheat seed development (NGDC accession: PRJCA034281) [39]. FPKM (fragments per kilobase of transcript per million mapped reads) values were used to quantify gene expression levels. Heatmaps were generated using TBtools.

### 2.10. CRISPR/Cas9-Mediated Generation of tagad1 Mutants

Using the recipient cultivar ‘Fielder’, which naturally lacks the TaGAD1-A homoeolog, mutations were introduced into TaGAD1-B and TaGAD1-D via CRISPR/Cas9, resulting in a 5-bp deletion and a 1-bp insertion, respectively. Consequently, a tagad1 triple mutant was obtained, with loss of function in all three homoeologs. Wild-type ‘Fielder’ plants were used as controls.

For salt tolerance phenotyping, 7-day-old WT and *tagad1* mutant seedlings grown hydroponically were treated with 0 mM (control) or 250 mM NaCl for 7 days. Phenotypic changes, including leaf wilting, chlorosis, and necrosis, were observed, recorded, and photographed daily. At the end of the treatment, shoot fresh weight and dry weight were measured as described in Section 2.9. The 250 mM concentration was chosen based on preliminary experiments to produce sufficiently pronounced morphological differences within the treatment period, enabling clear discrimination between the WT and mutant phenotypes.

### 2.11. Determination of GABA Content and GAD Enzyme Activity

Leaves (0.1 g) were harvested from 1-week-old WT and *tagad1* mutant plants grown under normal or salt-stressed conditions. GABA content and GAD enzyme activity were measured using the GABA Assay Kit and GAD Assay Kit (Su Zhou Grace Biotechnology Co., Ltd., Suzhou, China), respectively, according to the manufacturer’s instructions.

### 2.12. Histochemical Staining for Reactive Oxygen Species (ROS)

Hydrogen peroxide (H_2_O_2_) and superoxide anions (O_2_^−^) were detected using 3,3′-diaminobenzidine (DAB) staining and nitroblue tetrazolium (NBT) staining, respectively. Prior to staining, the seedlings were treated with 200 mM NaCl in half-strength Hoagland solution for 24 h. This concentration was selected to avoid staining saturation, as excessively high salt concentrations may trigger an overwhelming ROS burst that could mask genotype-specific differences.

For DAB staining, the samples were incubated with 1 mg/mL DAB solution (pH 3.8) for 18 h in the dark at 22 °C, and then decolorized in 95% (*v*/*v*) ethanol. For NBT staining, the leaves were fully immersed in 0.5 mg/mL NBT staining solution in 10 mM potassium phosphate buffer (pH 7.8) until a dark blue coloration developed, followed by decolorization in 95% (*v*/*v*) ethanol. The stained leaves were photographed to assess the accumulation of H_2_O_2_ and O_2_^−^.

### 2.13. Antioxidant Enzyme Activity Assays

To evaluate the antioxidant defense capacity of WT and *tagad1* mutant plants under salt stress, the activities of superoxide dismutase (SOD), peroxidase (POD), and catalase (CAT) were measured using the Superoxide Dismutase Activity Assay Kit, Peroxidase Activity Assay Kit, and Catalase Activity Assay Kit (COMIN), respectively, according to the manufacturer’s instructions. Total SOD activity was determined by measuring the percentage of inhibition of the pyrogallol autoxidation. Total POD activity was measured by monitoring the oxidation of 3,3′-dimethoxy-benzidine at 470 nm. Total CAT activity was assayed by measuring the rate of decomposition of H_2_O_2_ at 240 nm. All enzyme activities were calculated on a fresh weight (FW) basis and expressed as units per gram of fresh weight (U·g^−1^ FW). Each assay was performed with three biological replicates, and the average values were used for statistical analysis.

### 2.14. Statistical Analysis

All experiments were performed with at least three independent biological replicates. Data are presented as the mean ± standard deviation (SD). Statistical significance between the WT and *tagad1* mutant groups was determined using a two-sided Student’s *t*-test (*p* < 0.05, * *p* < 0.01). All statistical analyses were performed using GraphPad Prism (version 8.0; GraphPad Software, San Diego, CA, USA).

## 3. Result

### 3.1. Genome-Wide Identification of TaGADs Family Genes

The molecular weight (MW), theoretical isoelectric point (pI), instability index (II), aliphatic index, and subcellular localization were analyzed (Table S2). The predicted protein lengths ranged from 454 to 528 amino acids. MWs ranged from 51.76 to 59.62 kDa; TaGAD5-2A had the lowest MW, whereas TaGAD5-1A had the highest (Table S2). ProtParam analysis showed that the theoretical pI values ranged from 5.24 to 6.74. All TaGAD proteins were hydrophilic (GRAVY < 0), with aliphatic indices between 82.82 and 93.35. Most TaGAD proteins had instability index values below 40, indicating structural stability. All TaGAD proteins were predicted to localize to the cytoplasm.

Using the GAD amino acid sequences of Arabidopsis thaliana as references, we searched for *TaGADs* in the Triticum aestivum genome database using BLASTp. Redundant sequences were removed, and the remaining nonredundant sequences were verified for the presence of a GAD domain using the Pfam and SMART databases. A total of seven *TaGAD* genes, comprising 19 gene copies, were identified and designated *TaGAD1A–TaGAD6D* according to their chromosomal locations (Table S2). Notably, no homologous sequences corresponding to *TaGAD5-2B* or *TaGAD5-2D* were identified in the current wheat reference genome assembly [36].

### 3.2. Evolutionary Analysis

**Figure 1:**
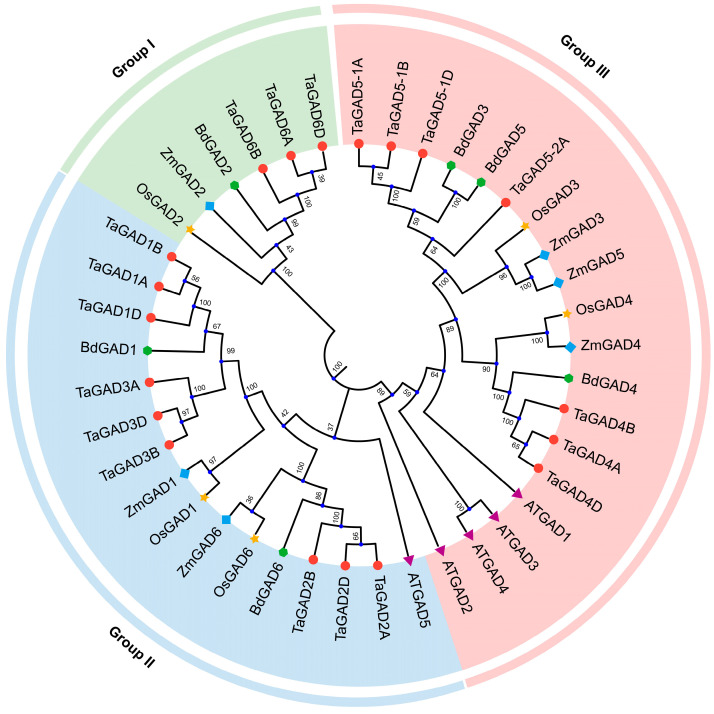
Evolutionary tree of *A. thaliana*, *O. sativa*, *T. aestivum*, and Zea mays GAD proteins. A neighbor-joining (NJ) phylogenetic tree was generated by MEGA12. The bootstrap values from 1000 replications are provided at each node. Red circles represent TaGAD (wheat), purple triangles represent AtGAD (*Arabidopsis*), yellow stars represent OsGAD (rice), blue squares represent ZmGAD (maize), and green hexagon represent BdGAD (*Brachypodium*).

To examine evolutionary relationships within the GAD family, multiple sequence alignments were performed using the 19 wheat homologs and GAD proteins from *Arabidopsis thaliana*, *Oryza sativa, Brachypodium distachyon*, and *Zea mays*. As shown in Figure 1, GAD proteins from different species were highly conserved and clustered into three subgroups. Group I included one BrGAD2, one OsGAD2, one ZmGAD2, and three wheat homologs: TaGAD6A, TaGAD6B, and TaGAD6D. Group II comprised seven wheat homologs (TaGAD5-1A, TaGAD5-1B, TaGAD5-1D, TaGAD5-2A, TaGAD4A, TaGAD4B, and TaGAD4D), four *A. thaliana* members (AtGAD1–AtGAD4), three *B. distachyon* members (BrGAD3–BrGAD5), two *O. sativa* members (OsGAD3 and OsGAD4), and three *Z. mays* members (ZmGAD3–ZmGAD5). Group III contained nine wheat homologs (TaGAD2A, TaGAD2B, TaGAD2D, TaGAD1A, TaGAD1B, TaGAD1D, TaGAD3A, TaGAD3B, and TaGAD3D), one *A. thaliana* member (AtGAD5), two *B. distachyon* members (BrGAD1 and BrGAD6), two *O. sativa* members (OsGAD1 and OsGAD6), and two *Z. mays* members (ZmGAD1 and ZmGAD6).

### 3.3. TaGAD Protein Motif and TaGAD Gene Structure

**Figure 2:**
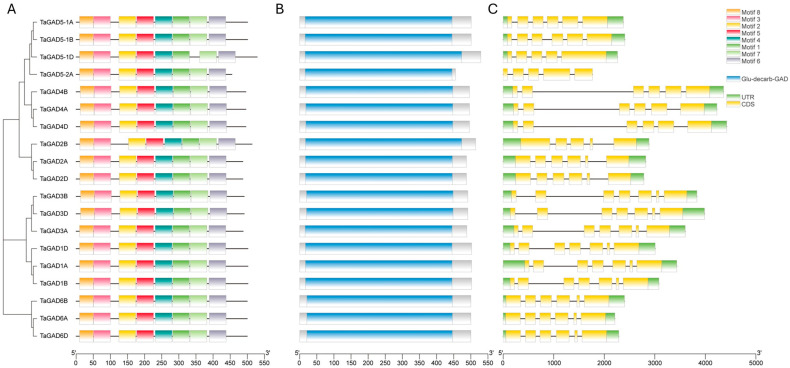
The motif distribution, conserved domains, and gene structures of *TaGAD* genes. The first column represents the motif distribution (**A**), the second column displays the conserved domain information (**B**), and the third column illustrates the gene structure information (**C**).

Gene structure analysis showed that the *TaGAD* genes contain five to seven exons and four to six introns. Eight conserved motifs were identified in the TaGAD proteins, and all 19 members shared these motifs, indicating a high degree of sequence conservation (Figure 2A). Conserved-domain analysis showed that all TaGAD proteins contained only a single Glu-decarb-GAD domain, suggesting a conserved structural core within this family (Figure 2B). Gene structure analysis revealed that TaGAD genes contained five to seven exons and four to six introns. Specifically, three genes contained five exons, ten contained six exons, and six contained seven exons (Figure 2C). Collectively, these findings suggest that TaGAD family members are highly conserved and may perform similar biological functions.

### 3.4. Prediction and Analysis of TaGAD Protein Structure

To further characterize TaGAD proteins, their structural features were analyzed. Spatial structure prediction (Table S3) showed that the secondary structures of TaGAD proteins are mainly composed of α-helices and random coils, with a small proportion of extended strands. No other secondary structure elements were detected. The tertiary structures were predicted using the SWISS-MODEL platform based on homology modeling.

**Figure 3:**
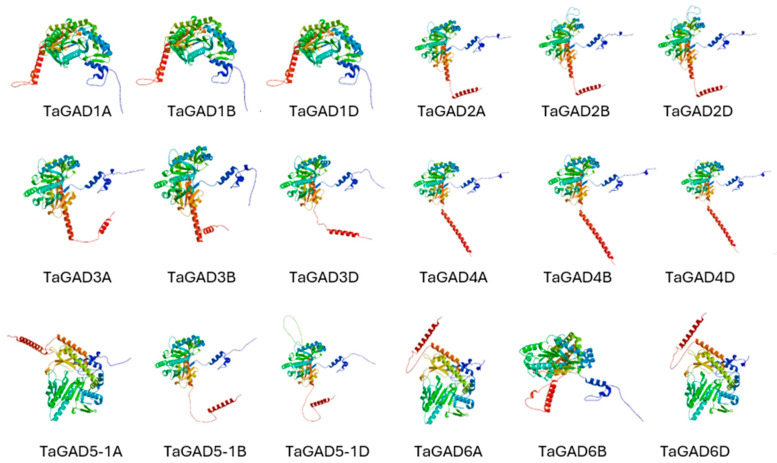
Predicted tertiary structures of TaGAD monomers generated by homology modeling. The TaGAD proteins share a conserved core domain, while variation is primarily observed in the terminal extensions. Most isoforms contain terminal α-helices consistent with putative calmodulin-binding domains, whereas TaGAD5-2A lacks these extensions.

Based on structural similarity, the GAD family proteins can be classified into four types. TaGAD2, TaGAD3, TaGAD4, TaGAD5-1B, and TaGAD5-1D form the first structural type with high similarity. TaGAD5-1A, TaGAD6A, TaGAD6B, and TaGAD6D form the second type, all characterized by extended terminal regions forming protruding α-helical arms. TaGAD5-2A, which lacks such terminal extensions and displays a compact conformation, forms the third type independently. TaGAD1 displays a distinct conformation and forms the fourth structural type independently (Figure 3).

### 3.5. Chromosome Localization and Collinearity Analysis of the TaGAD Gene Family

**Figure 4:**
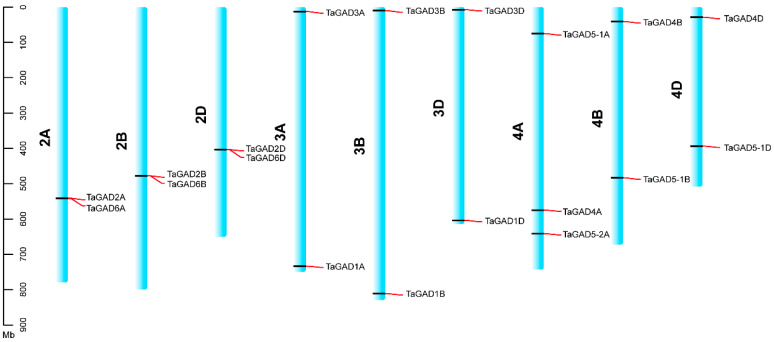
Chromosomal distribution of the TaGAD gene family in wheat. The 19 TaGAD genes are distributed across nine chromosomes (2A, 2B, 2D, 3A, 3B, 3D, 4A, 4B, and 4D). Blue bars indicate chromosomes, with chromosome numbers shown on the left and gene IDs on the right. The relative positions of TaGAD4 and TaGAD5 on chromosome 4A differ from those on 4B and 4D. Gene locations are based on IWGSC RefSeq v1.1 annotations.

Based on the chromosomal locations of *TaGAD* members, a chromosomal distribution map was generated (Figure 4). The 19 *TaGAD* genes are distributed across nine chromosomes: 2A, 2B, 2D, 3A, 3B, 3D, 4A, 4B, and 4D. TaGAD2 and *TaGAD6* (comprising six homologous genes) are clustered in close proximity on chromosomes 2A, 2B, and 2D, suggesting that these genes may have arisen from tandem duplication events. Second, *TaGAD5-1*, *TaGAD5-2*, and *TaGAD4* are located on the same chromosomes; however, the relative positions of TaGAD4 and TaGAD5 on chromosome 4A are reversed compared with those on chromosomes 4B and 4D. This is consistent with the known pericentric inversion that has uniquely reshaped chromosome 4A during the evolution of polyploid wheat [40,41]. Third, *TaGAD3* and *TaGAD1* are positioned near the distal ends of chromosomes 3 and 1, respectively, with considerable physical distances separating them. For this spatial distribution, although some members are located on the same chromosomes, they may have functionally diverged. Notably, *TaGAD2* (Group III) is located on a different chromosome from *TaGAD1* and *TaGAD3*, implying a possible convergent functional evolution of *TaGAD* genes across chromosomes.

**Figure 5:**
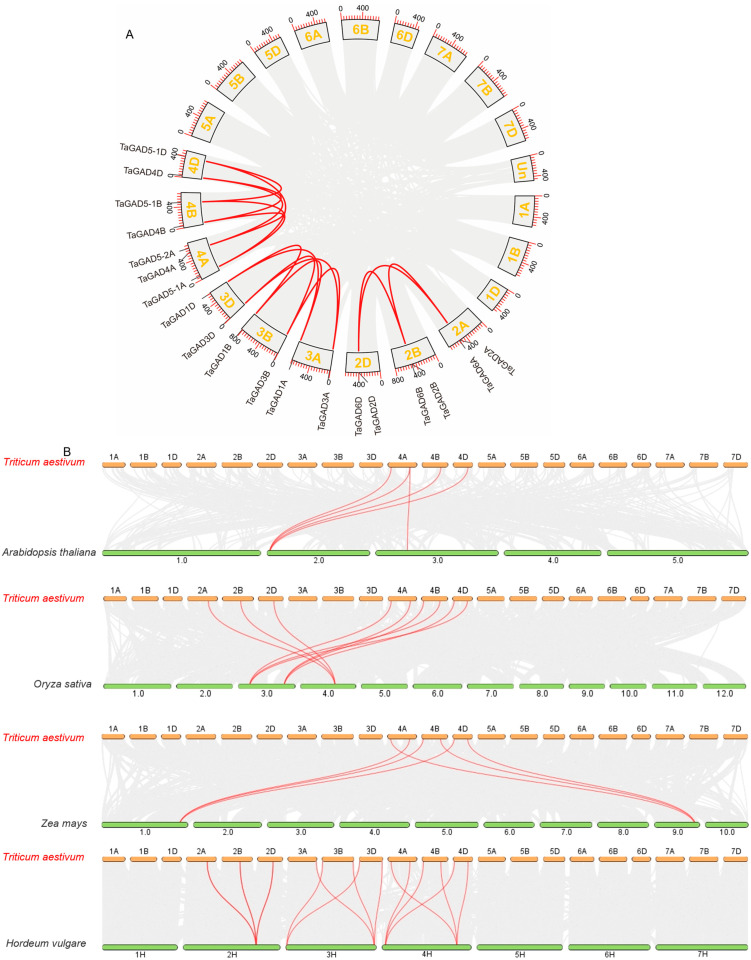
Collinearity analysis of GAD genes in *Triticum aestivum* (**A**). Note: The gray lines represent the collinearity relationships of all genes within the genomes of *Triticum aestivum*. The red lines represent the collinearity relationships of the GAD gene family across the genomes. Syntenic analysis of GAD genes between *Triticum aestivum*, *Arabidopsis thaliana*, *Oryza sativa*, *Zea mays*, and *Hordeum vulgare* (**B**). Gray lines in the background indicate the collinear blocks within *Triticum aestivum* and other plant genomes, whereas red lines highlight syntenic GAD gene pairs.

Collinearity analysis effectively reveals gene homology, and collinear homologs often retain similar functions. Therefore, TBtools was used to analyze and visualize collinearity within the *GAD* gene family. Intra-species collinearity analysis was conducted for TaGAD, followed by inter-species comparisons with other plants. Intra-species analysis identified collinear relationships among *TaGAD4*, *TaGAD5-1*, and *TaGAD5-2*; between *TaGAD1* and *TaGAD3*; and between *TaGAD2* and *TaGAD6*, indicating tandem and segmental duplication events (Figure 5A). These results suggest limited divergence of *TaGAD* genes during wheat evolution. Inter-species collinearity analysis with foxtail millet, the dicot model plant Arabidopsis thaliana, and monocot crops including rice, maize, and barley (Figure 5B) showed that *TaGAD5-1* and *TaGAD4* underwent segmental duplication across all four species, yielding 22 homologous gene pairs. These genes display close evolutionary relationships with their counterparts, indicating functional conservation. Additionally, *TaGAD2* exhibited segmental duplication in barley and rice, involving six homologous gene pairs. The nonsynonymous/synonymous substitution ratio (Ka/Ks) is commonly used to assess protein-level selection pressure. A Ka/Ks ratio > 1 indicates positive selection, a ratio of 1 indicates neutral evolution, and a ratio < 1 indicates purifying selection. Analysis of all 33 homologous gene pairs showed Ka/Ks ratios consistently below 1, suggesting that *TaGADs* have undergone purifying selection and have not acquired novel functions during evolution.

### 3.6. Analysis of Cis-Acting Elements in the TaGAD Gene Family Members

**Figure 6:**
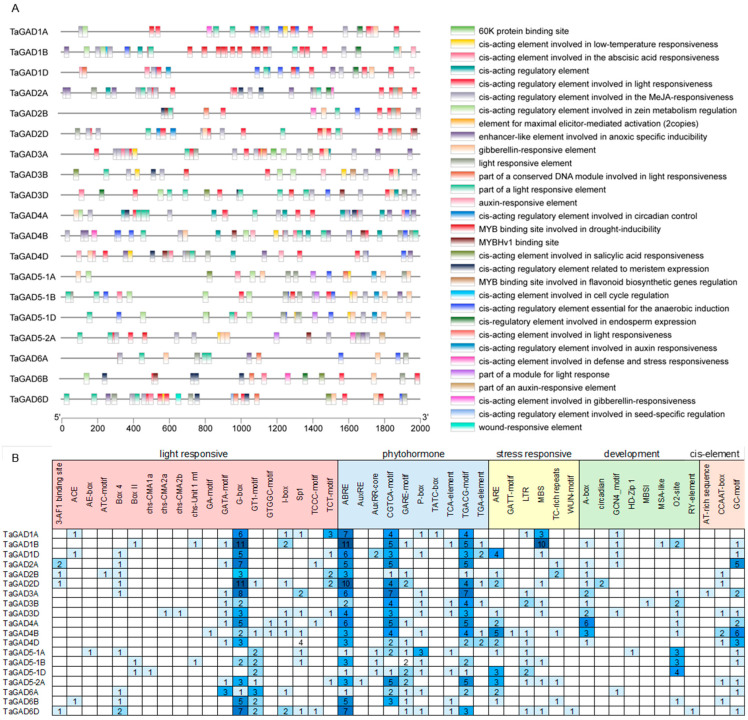
Cis-acting element analysis of TaGAD (**A**). Distribution of promoter cis-regulatory elements in light responsive, phytohormone, stress responsive, plant growth and development, and other cis-elements (**B**).

To further investigate the transcriptional regulatory mechanisms and potential functions of *TaGAD* gene family members, the 2000 bp upstream sequences from the start codon of each *TaGAD* gene were extracted and analyzed for cis-acting elements using the PlantCARE database (Figure 6A). As shown in the figure, the *TaGAD* promoters contain not only basic cis-acting elements, but also elements related to light responsiveness, phytohormone responses, stress responses, and growth and development (Figure 6B). Among the light-responsive elements, nearly 90% of *TaGAD* genes harbor G-box motifs. Phytohormone-responsive elements include the abscisic acid response element (ABRE); auxin-responsive elements (AuxRE, AuxRR-core, and TGA-element); gibberellin-responsive elements (P-box, TATC-box, and GARE-motif); the salicylic acid-responsive TCA-element; and methyl jasmonate (MeJA)-responsive elements (CGTCA-motif and TGACG-motif). All *TaGAD* genes contain ABREs, approximately 90% contain MeJA-responsive elements, and 63% contain gibberellin-responsive elements. Among the stress-responsive elements, 63% of *TaGAD* genes contain an ARE, and the *TaGAD1B* promoter contains 10 MBS motifs, indicating strong potential induction under drought stress (Figure 6B), findings that are consistent with previous studies [13]. Collectively, these results suggest that the *TaGAD* gene family in wheat is primarily regulated by phytohormones and is closely associated with stress and light responses.

### 3.7. Tissue-Specific and Abiotic Stress-Responsive Expression of TaGAD Genes

**Figure 7:**
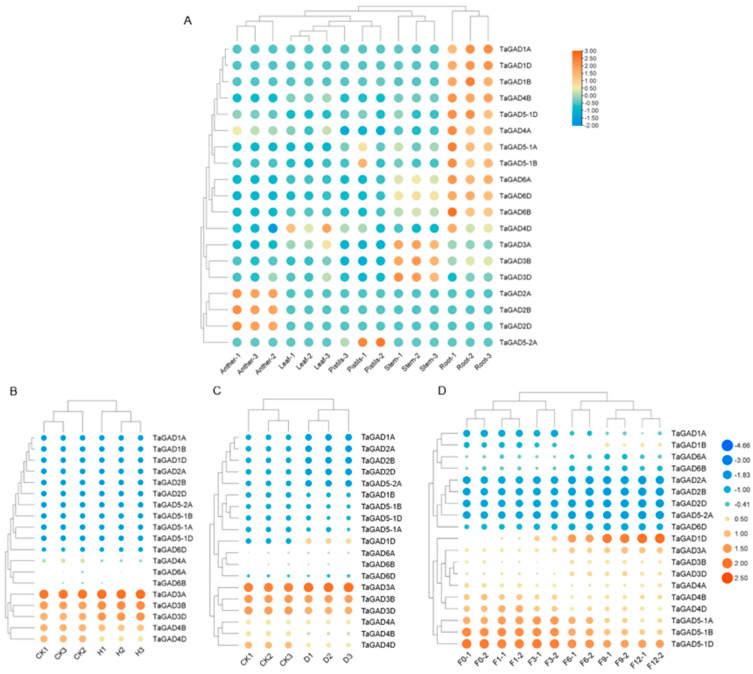
Expression patterns of TaGAD genes in different tissues and under abiotic stresses. (**A**) Tissue-specific expression profiles in root, stem, leaf, spike, and grain, derived from a publicly available transcriptome atlas (NGDC: PRJCA034281) [35]. FPKM values were used for quantification. (**B**) Expression changes under heat stress (42 °C) at 0 h and 6 h, as determined by RT-qPCR. (**C**) Expression changes under drought stress (air dehydration) at 0 h and 4 h, as determined by RT-qPCR. (**D**) Expression changes under salt stress (150 mM NaCl) at 0, 1, 3, 6, 9, and 12 h, as determined by RT-qPCR. RT-qPCR was performed with three biological replicates, and relative transcript levels were calculated using the 2^−ΔCT^ method.

To elucidate the expression patterns of *TaGAD* genes, we analyzed publicly available RNA-Seq data (https://ngdc.cncb.ac.cn/bioproject/browse/PRJCA034281) [39] to generate the tissue-specific expression profiles. Fragments per kilobase of transcript per million mapped read (FPKM) values were log_2_-transformed, and a clustered heatmap was generated to visualize the expression profiles of 19 *TaGAD* genes across different tissues (Figure 7A). *TaGAD1*, *TaGAD4*, *TaGAD5-1*, and *TaGAD6* showed similar patterns, with high expression in roots. *TaGAD3* was predominantly expressed in stems, whereas *TaGAD2* exhibited strong expression in anthers and low expression in other tissues. This result is consistent with previous reports that GAD2 is specifically expressed in spikes and further refines its localization to anthers within the spike. In contrast, *TaGAD5-2A* displayed a distinct pattern, with high expression in pistils, suggesting the potential functional divergence of *TaGAD* genes during wheat growth and development.

To determine whether the *TaGAD* gene family responds to abiotic stress, we examined the expression patterns of *GAD* family members under heat, drought, and salt stress by RT-qPCR. The results showed that after heat stress, the *TaGAD* family exhibited distinct response patterns, with *TaGAD3A*, *TaGAD3B*, and *TaGAD3D* being significantly upregulated, whereas the homoeologous copies of *TaGAD4* were downregulated. The expression levels of the remaining genes did not change markedly under heat stress (Figure 7B). We then analyzed the expression patterns of the *TaGAD* family under drought stress, revealing that all genes except *TaGAD4* were upregulated (Figure 7C). On this basis, we further examined the dynamic expression patterns under salt stress. The results indicated that all *TaGAD* family genes responded to salt stress, with *TaGAD4* and *TaGAD5* exhibiting gradually decreasing expression levels over the duration of salt stress, while *TaGAD1*, *TaGAD6*, *TaGAD2*, and *TaGAD3* showed gradually increasing expression levels. Notably, *TaGAD1D* displayed the most pronounced upregulation with prolonged salt stress (Figure 7D), suggesting that *TaGAD1* may be involved in the regulation of salt stress responses.

We further examined whether the abundance of ABRE and MBS motifs in the *TaGAD* promoters (identified in Section 3.6) correlated with the stress-induced expression patterns described above. No strict correlation was observed between the copy numbers of these elements and the expression levels under drought or salt stress. For example, *TaGAD1D*, which contains only one MBS motif, exhibited a higher fold change under drought stress than *TaGAD1B*, which harbors ten. Similarly, genes lacking MBS elements altogether displayed divergent responses: some were downregulated (*TaGAD2A*, *TaGAD2B*, *TaGAD4A*, *TaGAD4B*, *TaGAD4D*), others showed no significant change (*TaGAD6A*, *TaGAD6B*), while a few were still induced (*TaGAD3A*, *TaGAD5-1A*). These observations indicate that the presence of ABRE and MBS elements alone does not reliably predict the stress-responsive behavior of individual *TaGAD* genes.

### 3.8. TaGAD1 Positively Regulates Salt Tolerance Through GABA-Mediated ROS Detoxification

**Figure 8:**
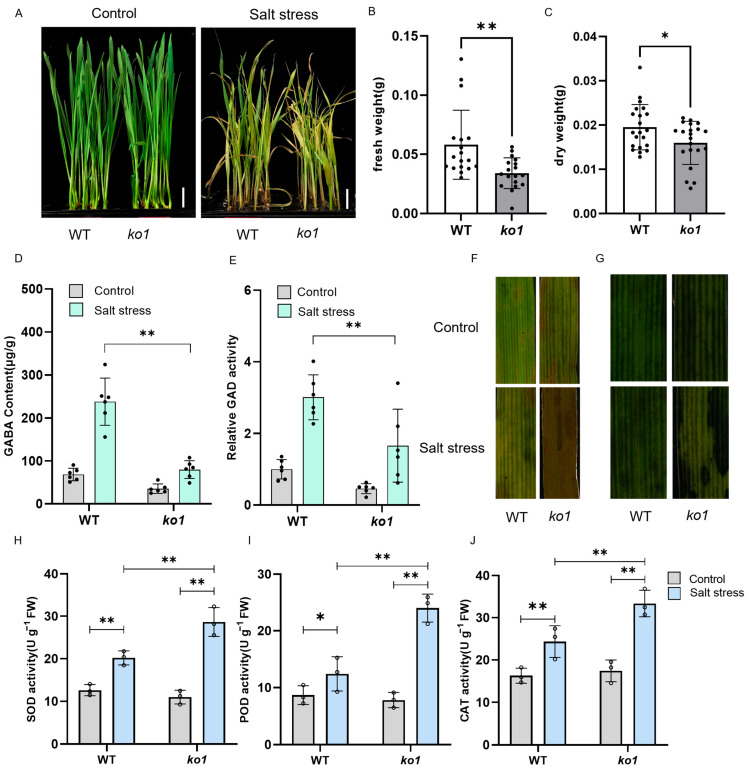
TaGAD1 positively regulates the salt tolerance of wheat. (**A**) Salt stress tolerance assay of *tagad1* mutant and wild-type plants. Scale bars, 2 cm. (**B**,**C**) Fresh weight and dry weight of *tagad1* mutant and wild-type plants after salt stress. (**D**,**E**) GABA content [14] and relative GAD activity in *tagad1* mutant and wild-type plants under normal conditions and salt stress treatment. (**F**,**G**) Histochemical staining of NaCl-induced ROS production in leaves of WT Fielder and tagad1 ko lines under control or NaCl treatment visualized using NBT (**F**) or DAB staining (**G**). (**H**–**J**) The activities of SOD, POD, and CAT in wheat seedling leaves, respectively. * *p* ≤ 0.05; ** *p* ≤ 0.01.

To elucidate the role of *TaGAD1* in wheat salt tolerance, we performed salt tolerance assays using CRISPR/Cas9 mutant lines generated in a previous study [13]. Under salt stress, both wild-type (WT) and mutant plants were evaluated phenotypically. The *tagad1* mutants exhibited more severe leaf wilting and chlorosis than WT plants (Figure 8A), indicating a marked reduction in salt tolerance. Although the mutants showed pronounced growth inhibition under salt stress, they remained green and did not exhibit complete necrosis. Therefore, fresh and dry weights were used as the primary indicators of seedling salt tolerance. Consistently, both fresh and dry weights were significantly lower in the mutants than in WT plants (Figure 8B,C), confirming that knockout of *TaGAD1* markedly impairs salt tolerance in wheat.

To explore the possible causes of the differential salt tolerance, we measured GABA content and GAD activity in the leaves of WT and mutant plants under both normal and salt-stressed conditions. The results showed that GABA content and GAD activity were significantly lower in the mutants compared with the WT under both conditions (Figure 8D,E), suggesting that *TaGAD1* knockout compromises GABA accumulation and GAD enzymatic function.

To determine whether the reduced GAD activity and GABA content in the mutants affect ROS scavenging, we used 3,3′-diaminobenzidine (DAB) and nitro blue tetrazolium (NBT) staining to detect H_2_O_2_ and O_2_^−^ levels, respectively, in the leaves. The results showed that H_2_O_2_ and O_2_^−^ levels were higher in *tagad1* mutant leaves compared with the WT (Figure 8F,G). In contrast, the activities of the antioxidant enzymes SOD, POD, and CAT were significantly lower in the mutants than in the WT. Collectively, these results demonstrate that knockout of *TaGAD1* reduces GABA accumulation and GAD activity, which in turn attenuates the ROS scavenging capacity mediated by antioxidant enzymes. This impairment leads to excessive ROS accumulation and oxidative stress, ultimately resulting in decreased salt tolerance in wheat.

## 4. Discussion

This study presents the first genome-wide identification of the *TaGAD* gene family in hexaploid wheat, identifying seven members with 19 gene copies distributed unevenly across nine chromosomes (2A, 2B, 2D, 3A, 3B, 3D, 4A, 4B, 4D). This number exceeds those reported in diploid species such as *A. thaliana* (5 members), rice (6), and maize (6), consistent with the genomic complexity of allohexaploid wheat. The expansion of the *TaGAD* family was driven by two polyploidization events [41,42,43,44], with most genes retained as triads (A/B/D subgenomes), as illustrated by *TaGAD1A/1B/1D* and *TaGAD2A/2B/2D*. Notably, no homologous sequences corresponding to TaGAD5-2B or TaGAD5-2D were detected in the current wheat reference genome assembly, suggesting possible gene loss, structural variation, or incomplete assembly in the corresponding subgenomes. This pattern resembles the expansion of the *GIF* and *CHS* gene families in soybean, which was driven by whole-genome duplication [45,46]. Chromosomal localization and collinearity analyses further showed that *TaGAD* family expansion mainly resulted from segmental duplication, while the close proximity of *TaGAD2* and *TaGAD6* on chromosomes suggests tandem duplication. These duplication events increased the family size and provided a basis for potential functional divergence.

Ka/Ks analysis showed that all *TaGAD* homologous gene pairs had Ka/Ks ratios < 1, indicating that the family has primarily evolved under purifying selection, which implies strong functional constraints on this key metabolic enzyme. Similar patterns of high conservation have been reported for the *CHS* and *GIF* families [45,46], suggesting that GAD, as a key metabolic enzyme, is under strong functional constraint during evolution. Despite this overall conservation, some homologous genes (e.g., *TaGAD5-1A* and *TaGAD5-2A*) differ in protein length, isoelectric point, and tertiary structure, indicating possible variation in substrate affinity, calmodulin-binding capacity, or subcellular localization. The evolutionary tree further grouped TaGADs into three subfamilies, with strong cross-species collinearity within each subfamily, supporting their evolutionary conservation.

Gene structure analysis showed that all *TaGAD* members contain 5–7 exons, with similar exon–intron patterns within each subfamily. All TaGAD proteins contain a complete Glu-decarb-GAD domain, and eight conserved motifs are retained across all 19 homologous genes, highlighting strong structural conservation. Similar patterns have been reported in the CHS, GIF, and ALDH families [45,46,47], where genes within the same subfamily share similar gene structures and motif compositions. Tertiary structure prediction further classified the TaGAD family into four conformational types, with TaGAD1 showing a distinct spatial folding pattern, suggesting specialized functions or regulatory roles. Together with predictions of cytoplasmic subcellular localization for all members, these findings imply that TaGADs likely function by modulating GABA metabolism, protein interactions, and signal transduction within the cytoplasm.

The structural classification of TaGAD proteins into four types based on tertiary structure prediction is intriguing, as it raises the possibility of functional divergence among family members. Notably, the presence or absence of extended terminal α-helical regions appears to correlate with potential regulatory modes. In plant GADs, the C-terminal extension is known to contain a calmodulin (CaM)-binding domain that mediates the calcium-dependent regulation of GAD activity [23,48]. Therefore, members possessing such extensions (TaGAD5-1A, TaGAD6A, TaGAD6B, and TaGAD6D) may be subject to Ca^2+^/CaM-dependent regulation, whereas TaGAD5-2A, which uniquely lacks this extension, may function in a CaM-independent manner. However, experimental validation—such as CaM-binding assays and activity measurements under different Ca^2+^ concentrations—is required to confirm this hypothesis. Nevertheless, these structural distinctions provide a useful framework for predicting potential functional specialization within the TaGAD family.

*Cis*-element analysis of the *TaGAD* promoters revealed a diverse array of elements associated with hormone responses, abiotic stress, and light signaling. The universal presence of ABRE (ABA-responsive) and CGTCA/TGACG (MeJA-responsive) elements across all members suggests that the *TaGAD* family may contribute to abiotic stress tolerance through abscisic acid and jasmonate signaling pathways. The enrichment of stress-responsive elements (ARE, MBS) and hormone-responsive elements is consistent with findings in sorghum *SbJAZ* genes [49] and cauliflower *BoRR* genes [50], highlighting the conserved role of hormone signaling in regulating stress-responsive gene families. Additionally, the *TaGAD1B* promoter uniquely contains 10 MBS elements, indicating a potential specialized role in drought stress response. As calmodulin-binding proteins, *TaGAD* genes also universally contain G-box light-responsive elements, suggesting possible crosstalk between calcium signaling and light signaling in stress adaptation, a mechanism that remains underexplored and requires further investigation. Intriguingly, however, when we examined the relationship between MBS and ABRE motif abundance and the stress-induced expression patterns of individual *TaGAD* members, no strict correlation was observed. For instance, *TaGAD1D*, which contains only one MBS motif, exhibited a higher fold change under drought stress than *TaGAD1B*, which harbors ten. Moreover, genes lacking MBS elements altogether displayed divergent responses: some were downregulated (*TaGAD2A*, *TaGAD2B*, *TaGAD4A*, *TaGAD4B*, *TaGAD4D*), others showed no significant change (*TaGAD6A*, *TaGAD6B*), while a few were still induced (*TaGAD3A*, *TaGAD5-1A*). These observations suggest that the presence or absence of a single type of cis-element does not definitively determine stress responsiveness. We speculate that the combinatorial arrangement of multiple cis-elements, the availability and activity of specific transcription factors, and the positional distribution of these elements within the promoter may collectively modulate the transcriptional output under specific stress conditions. However, direct evidence for these possibilities is currently lacking, and further experimental validation—such as promoter deletion or mutation analyses—would be required to dissect the precise regulatory logic governing *TaGAD* expression under stress conditions.

Based on public transcriptome data and qRT-PCR validation, *TaGAD* family members show differential expression across tissues. *TaGAD1, TaGAD4, TaGAD5-1*, and *TaGAD6* are highly expressed in roots; *TaGAD3* is mainly expressed in stems; TaGAD2 shows anther-specific expression; and *TaGAD5-2* is enriched in pistils. This divergence resembles the tissue-specific expression of *GmGIF* genes in soybean, suggesting that homologous genes may have undergone sub-functionalization or neo-functionalization after duplication events, Notably, the anther-specific expression of *TaGAD2* aligns with its distinct phylogenetic placement in Group I, implying a specialized role in reproductive organ development or pollen wall formation, similar to the role of CHS-like proteins LAP5/LAP6 in pollen exine synthesis in Arabidopsis [45].

The present study demonstrates that knockout of TaGAD1 leads to a severe salt-sensitive phenotype in wheat seedlings under salt stress, characterized by exacerbated growth inhibition, significantly reduced GABA accumulation and GAD activity, excessive accumulation of reactive oxygen species (ROS), and decreased antioxidant enzyme activities. These findings collectively confirm that *TaGAD1* acts as a positive regulator of salt tolerance in wheat.

Our results demonstrated that both GABA content and GAD activity were significantly lower in *tagad1* knockout lines under salt stress, confirming that TaGAD1 participates in salt tolerance regulation through the GABA metabolic pathway. As the key rate-limiting enzyme of the GABA shunt, GAD catalyzes the irreversible decarboxylation of glutamate to produce GABA [18]. Accumulating evidence indicates that GABA functions as a signaling molecule that enhances antioxidant defense systems and regulates ion homeostasis, thereby improving salt stress tolerance [20]. In Arabidopsis, GABA-deficient mutants display increased salt sensitivity, and the underlying mechanism involves the GABA-mediated regulation of H^+^-ATPase activity, reduced K^+^ efflux, and the upregulation of SOS1 and NHX1 expression [22]. Furthermore, GAD activity is tightly regulated by cytosolic Ca^2+^ concentration and calmodulin (CaM) [23], and Ca^2+^ signaling plays a central role in the early transient responses of plants to salt stress [51]. Therefore, we propose that TaGAD1-mediated GABA metabolism cross-talks with the Ca^2+^ signaling and ROS homeostasis regulatory networks to influence salt stress adaptation in wheat. A recent study showed that exogenous GABA enhances wheat salt tolerance via ion homeostasis and photosynthetic protection, and that TaGAD6 silencing compromises this tolerance [24]. However, the role of TaGAD1 in salt tolerance and its mechanistic link to ROS detoxification remain unexplored.

ROS homeostasis plays a critical role in *TaGAD1*-mediated salt tolerance. Our results show that H_2_O_2_ and O_2_^−^ levels are significantly elevated in *tagad1* mutants, demonstrating excessive ROS accumulation. Consistent with this, the activities of the antioxidant enzymes SOD, POD, and CAT were significantly reduced in the mutants compared with the WT. This indicates that loss of *TaGAD1* impairs the ROS scavenging system at the enzymatic level, which, together with the reduction in GABA-mediated detoxification, leads to the disruption of ROS homeostasis. This finding aligns with previous reports that GABA positively regulates antioxidant enzyme activities under stress conditions; for example, exogenous GABA application has been shown to enhance the activities of SOD, POD, and CAT in multiple plant species subjected to salt stress [22,23]. Similarly, other salt tolerance regulators have been shown to influence ROS metabolism; for example, the histone acetyltransferase TaHAG1 enhances wheat salt tolerance by targeting respiratory burst oxidase homolog (Rboh) genes to regulate ROS production [52]. While TaHAG1 acts primarily at the level of ROS generation, our findings suggest that TaGAD1 participates in the fine-tuning of ROS metabolism through GABA-mediated ROS scavenging capacity, highlighting a complementary regulatory mechanism. Thus, the ion/photosynthetic protection conferred by TaGAD6 and the ROS detoxification mediated by TaGAD1 may represent complementary mechanisms of GABA-mediated salt tolerance in wheat [24].

Taken together, we propose a working model in which TaGAD1 positively regulates salt tolerance in wheat by mediating GABA accumulation, which in turn promotes ROS detoxification through the activation of antioxidant enzymes. This regulatory axis appears to intersect with Ca^2+^ signaling, given the Ca^2+^/CaM-dependent regulation of GAD activity. However, several key questions remain to be addressed; in particular, how GABA is perceived and how this signal is transduced to modulate the activities of ROS-scavenging enzymes are not yet understood. The receptor(s) or sensor(s) for GABA in plants, as well as the downstream signaling components connecting GABA accumulation to antioxidant enzyme activation, have yet to be identified. Addressing these questions will be essential for a comprehensive understanding of the TaGAD1–GABA–ROS regulatory pathway.

To validate the above hypotheses, future investigations could focus on the following aspects: (i) metabolite and gene expression analysis—systematically quantify the levels of GABA and its upstream and downstream metabolites in *tagad1* mutants using LC-MS to complement the initial GABA measurements, and analyze the expression of ion transporter genes (e.g., *SOS1, NHX1, HKT1*) and key Ca^2+^ signaling genes under salt stress to establish a transcriptional link between TaGAD1 and ion homeostasis and signal transduction; (ii) exogenous complementation and protein–protein interaction assays—exogenously apply GABA to the *tagad1* mutant to test whether it can rescue the salt-sensitive phenotype, and employ yeast two-hybrid or co-immunoprecipitation assays to explore direct interactions between TaGAD1 and CaM or ion transporters; (iii) transcriptome sequencing—perform RNA-Seq on wild-type and *tagad1* mutant plants under salt stress to systematically identify differentially expressed genes regulated by TaGAD1, focusing on pathways related to ROS metabolism, Ca^2+^ signaling, and ion transport, thereby constructing a downstream regulatory network.

In summary, this study demonstrates that *TaGAD1* positively regulates salt tolerance in wheat by maintaining GABA accumulation and GAD activity, which are essential for sustaining ROS scavenging capacity and ROS homeostasis. Our findings establish a molecular framework for understanding GABA-dependent salt stress adaptation and highlight *TaGAD1* as a promising target for molecular breeding to improve wheat salt tolerance.

## 5. Conclusions

This study systematically identified the *GAD* gene family in hexaploid wheat, revealing seven *TaGAD* genes comprising 19 gene copies with strong evolutionary conservation and stress-responsive cis-elements in their promoters. Functional characterization demonstrated that CRISPR/Cas9-mediated knockout of *TaGAD1* significantly reduced salt tolerance, accompanied by decreased GABA content and GAD activity, as well as excessive ROS accumulation. These results demonstrate that *TaGAD1* acts as a positive regulator of salt tolerance in wheat through maintaining GABA-mediated ROS homeostasis, providing direct genetic evidence for this mechanism. Our findings establish a molecular framework for understanding GABA-dependent salt stress adaptation and highlight *TaGAD1* as a promising target for molecular breeding to improve wheat salt tolerance.

## Data Availability

The original contributions presented in the study are included in the article and Supplementary Materials. Further inquiries can be directed to the corresponding author.

## Supplementary Materials

The following supporting information can be downloaded at: https://www.mdpi.com/article/10.3390/plants15152281/s1, Table S1: RT-qPCR primers for wheat TaGAD gene family members; Table S2: Main characteristics of wheat TaGAD proteins family members; Table S3: Secondary structure of wheat TaGAD proteins family members.
